# Anti-Inflammatory Properties and Gut Microbiota Modulation of *Porphyra tenera* Extracts in Dextran Sodium Sulfate-Induced Colitis in Mice

**DOI:** 10.3390/antiox9100988

**Published:** 2020-10-14

**Authors:** Jungman Kim, Jae Ho Choi, Gwangpyo Ko, Hyejun Jo, Taehwan Oh, Byungjae Ahn, Tatsuya Unno

**Affiliations:** 1 Faculty of Biotechnology, School of Life Sciences, SARI, Jeju National University, Jeju 63243, Korea; kjm5364@gmail.com (J.K.); rhkdvy1004@gmail.com (G.K.); hyejun758@gmail.com (H.J.); 2 Subtropical/Tropical Organism Gene Bank, Jeju National University, Jeju 63243, Korea; jaehochoi78@gmail.com; 3 Marine Biotechnology Research Center, Jeonnam Bioindustry Foundation, Wando 59108, Korea; sosoth2@hanmail.net (T.O.); chemeditech@hanmail.net (B.A.)

**Keywords:** *Porphyra tenera*, colitis, gut microbiota, inflammation

## Abstract

*Porphyra tenera* (PT) is a functional seaweed food that has been reported for health benefits such as antioxidant, immunostimulant, anti-inflammation, and hepatoprotective effects. In this study, we investigated the effect of PT extracts on gut microbiota modulation in colitis-induced mice. The mice experiment was designed as three groups including normal mice (CTL), dextran sodium sulfate (DSS)-fed mice, and DSS plus PT extracts-fed mice (PTE). DSS was administrated through drinking water containing DSS for 1 week, and the PT extract was ingested into the gastrointestinal tract in mice. PT extract ameliorated the decreased body weight and colon length and improved disease activity index and pro-inflammatory cytokine expression. In addition, PT extract significantly shifted the gut microbiota of mice. DSS treatment significantly increased the portion of harmful bacteria (i.e., *Helicobacter*, *Mucipirillum*, and *Parasutterella*) and decreased the butyrate producing bacteria (i.e., *Acetatifactor*, *Alistipes*, *Oscillibacter*, and *Clostridium_XIVb*). PT extract increased the abundance of genera *Clostridium_XIVb* and also enriched some of predicted metabolic activities such as glyoxylate cycle, ethylmalonyl-CoA pathway, nitrate reduction, creatinine degradation, and glycine betaine metabolism. These results suggest that PT extract may ameliorate the DSS-induced colitis inflammation through regulating the compositions and functions of gut microbiota in mice.

## 1. Introduction

Recent studies have revealed that the balance of the intestinal microbial environment plays an important role in controlling inflammation and oxidative damage in the colon. It has been found that the composition of intestinal micro-organisms and microbial metabolites is significantly altered in inflammatory colitis patients and colitis-induced mouse models [1]. Furthermore, gut microbial diversity is important in linking diet to host physiology and pathology, which is influenced by dietary composition and pattern [2]. The gut microbiota is a complex ecosystem that plays a critical role in regulation of immune system homeostasis. Recent studies showed that gut microbes are related with many diseases such as obesity, type 2 diabetes, non-alcoholic fatty liver disease, cardiovascular disease, nervous system disease, and IBD [3,4,5,6,7]. It has been reported that dysbiosis of gut microbiota exacerbate the development of these diseases. Some studies reported the interaction between the gut bacteria and the mucosal immune system that plays an important role in maintaining intestinal health and preventing the development of IBD [8].

Seaweeds are composed of approximately 70% polysaccharides. Seaweeds contain indigestible dietary fibers for humans and only digested or fermented by intestinal microbes in the large intestine. The final digested products, such as short chain fatty acids, are absorbed to the host’s epithelial cells and control the host immune system [9]. Among the edible seaweeds, *Porphyra* has been a diet consumed in Korea, over a long period of time. The dried form of *Porphyra* contains approximately 35% water-soluble polysaccharides, including 10% porphyran [10]. Many studies have reported that *Porphyra tenera* (PT) extracts is the rich in total polyphenols and total flavonoids, which have antioxidant and radical scavenging activities [11]. Porphyran, a major component of PT, inhibits ear edema in mice by 2,4,6-trinitrochlorobenzene-induced contact hypersensitivity [12]. The volatile oil of PT, which has a bioactive chemical components and strong antioxidant effect, can be used in the food industry as food additive as well as a cosmetic duet to its antioxidant properties [13]. Mycosporine-like amino acids extracted from PT protect skin from UV irradiation-induced photodamage [14]. Furthermore, PT possesses chemo-preventive effects against diethylnitrosamine-induced hepatocarcinogenesis [15]. Previous studies have reported that PT extracts enhanced the immune response through NF-κB immunostimulant in macrophage [16]. Furthermore, PT extracts supplementation enhanced immune function by improving natural killer cell activity without adverse effects in healthy adults [17].

Although PT extracts has been reported to exhibit various beneficial effects, its efficacy against inflammatory colitis and gut microbiota dysbiosis has not been investigated. In this study, we evaluated the inhibitory effects of PT extracts on intestinal inflammation and gut microbiota in dextran sulfate sodium (DSS)-induced colitis mice. Administration of DSS induces mucosal damage, which shifts the gut microbiota and brings about changes in cytokine levels and other physical characteristics such as length of intestines and body weight.

## 2. Materials and Methods

### 2.1. Preparation of Porphyra tenera (PT) Extracts

PT extracts used in this study were previously characterized [17]. PT were collected from the coastal area of the island (Soan-do; 38°8′29.36″ N, 126°39′22.59″ E) in the southern coast of Korea in February and March, 2015 (Figure S1), repeatedly washed with fresh water and then air-dried at 50 °C for 72 h. Extraction was performed at 80 ± 2 °C for 3 h using 10% ethanol (*v*/*v*). PT extracts was freeze-dried after filtration using a 1 µm housing filter. The extract was concentrated to approximately 10–20 Brix at 65–70 °C. The composition of PT extracts and the concentration of porphyra-334 in PT extracts were described in our previous study [17]. PT extracts was stored at −20 °C until used in animal experiment.

### 2.2. Determination of Total Polyphenols Content

Total polyphenol content was measured by Folin-Ciocalteu assay [18]. Sample solution (300 µL) was mixed with Folin-Ciocalteau (160 µL) and dilution water (250 µL) for 5 min. Then, a 10% sodium carbonate solution (300 µL) was added to the reaction and the solution was incubated at room temperature for 30 min. Optical density was determined at 750 nm using a VERSA max microplate reader (Molecular Device, San Jose, CA, USA). Results were calculated in mg gallic acid equivalent (GAE)/g sample.

### 2.3. Determination of 1,1-Diphenyl-2-Picrylhydrazyl (DPPH) Free Radical Scavenging Activity

Free radical scavenging ability of the PT extracts was evaluated by DPPH radical scavenging assay as previously reported with a minor modification [19]. The 0.1 mM DPPH solution was diluted with 150 µL of ethanol and added to 50 µL of the sample. The reaction mixture was vortexed and left in the dark for 30 minutes at room temperature. The optical density was determined using a VERSA max microplate reader with a wavelength at 515 nm (Molecular Device, San Jose, CA, USA). Results were calculated using the following equation. Ac and As is the absorbance of the control and sample.

DPPH radical scavenging capacity (%) = [(Ac − As)/Ac] × 100(1)

### 2.4. Animal Experiment

Specific pathogen-free 6-week-old male BALB/c mice were obtained from DBL (Eumseong, Korea). The mice were acclimatized for 1 week prior to use and were housed in an air-conditioned room with a 12 h light/dark cycle at a temperature of 22 ± 2 °C with 50 ± 5% relative humidity. All experimental protocols for animal care were performed according to the rules and regulations of the Animal Ethics Committee of Jeju National University (the Institutional Animal Care and Use Committee of Jeju National University; Approval number 2018-0039) and conducted according to the Korean Animal and Plant Quarantine Agency guidelines (Laboratory Animal Guideline-75).

First, we investigated the effect of the immune system by PT extracts in mice. After acclimatization, the mice were separated for 3 groups (10 mice per group) according to body weight. The groups include Control group (no PT extracts treated), PT extracts-L (500 mg/kg PT extracts-treated group), and PT extracts-H (1000 mg/kg PT extracts-treated group) as previously described [20]. PT extracts dissolved in normal saline and mice were intragastrically administered with PT extracts once a day for 28 days. The mice spleen was collected for immunological evaluations after sacrificed by CO_2_ inhalation. The distribution of immune cells including B cells and T cells in splenocytes isolated from mice fed with PT extracts was evaluated by fluorescence-activated cell sorting (FACS) analysis.

Second, we investigated the effect of PT extracts on 4% dextran sulfate sodium (DSS) (MP Biomedicals Inc., Irvine, CA, USA)-induced colitis in mice. Accordingly, we divided the mice into 3 groups by body weight: Control (normal group, *n* = 10); DSS (DSS-treated group, *n* = 10); PTE (DSS plus PT extracts-treated group, *n* = 10) (Figure S2A). Fecal samples were collected from each mouse at day 0 and 7. At the end of the experiment (Day 7), the mice were sacrificed by rodent carbon dioxide (CO_2_) euthanasia according to AVMA guidelines for the Euthanasia of Animals and the colon tissues were stored under −80 °C.

### 2.5. Preparation of Splenocytes and Fluorescence-Activated Cell Sorting (FACS) Analysis

Splenocytes were obtained by gentle disruption of the spleen through sieve mesh. After the lysis of spleen tissues with 1× RBC Lysis Buffer (Roche, Basel, Switzerland), remaining cells were re-suspended in RPMI-1640 medium with 10% fetal bovine serum, 100 µg/mL streptomycin and 100 IU/mL penicillin, followed by centrifugation and washing with phosphate buffered saline (WELGENE, Gyeongsan, Korea).

FACS was used to analyze sub types of the isolated splenocytes. Briefly, spleen cells were harvested and washed with 1× Dulbecco’s Phosphate Buffered Saline (DPBS, WELGENE, Gyeongsan, Korea). The cells were blocked with 1 µg anti-mouse IgG solution in PBS for 15 min at 4 °C, and then stained with fluorescently labeled monoclonal antibodies (Biolegends, San Diego, CA, USA) for an additional 15 min at 4 °C. Monoclonal anti-bodies were directly labeled with the following fluorescent tags; CFSE for Live/Dead cell, PE-CD3 for total T cell, APC-CD4 for helper T cell, Pacific-Blue-CD8a for cytotoxic T cell and regulatory T cell, and PE/Cy7-CD45R/B220 for B cell. After centrifugation, DPBS was added to the cells, and twenty thousand viable cells per treatment (as determined by light scatter profiles) were analyzed using a BD FACS LSR Fortessa flow cytometer (BD Biosciences, Franklin Lakes, NJ, USA).

### 2.6. Disease Activity Index

Disease activity index (DAI) scores were calculated as described in a previous study [21]. Body weight loss, stool consistency, and fecal bleeding were observed at the end of the experiment. Stool consistency was evaluated by comparing with the feces obtained from the Control. Fecal bleeding was observed using EZ detect colon disease test (BIOMERICA Inc., Irvine, CA, USA). The DAI score for these 3 factors were calculated as follows: weight loss (0, none; 1, 1–5%; 2, 5–10%; 3, 10–20%; and 4, over 20%); stool consistency (0, well form pellets; 2, loose stool; and 4, diarrhea) and fecal bleeding (0, negative; 2, positive; and 4, gross bleeding).

### 2.7. RNA Extraction and Real-Time PCR

Total RNA was extracted from colon tissues using RNAiso Plus reagent (TaKaRa Bio. Inc., Shiga, Japan). Total RNA concentration was verified using a spectrophotometer, DS-11 plus (DENOVIX Inc., DE, USA). cDNA was synthesized from 1 µg of RNA using PrimeScript^TM^ 1st strand cDNA Synthesis Kit (Takara Bio Inc., Shiga, Japan), and oligo dT primer was used for reverse-transcription. For quantitative analysis of cytokines using real-time PCR, 1 µL of cDNA was used to amplify cytokine genes, such as TNF-α, IL-6, IL-1β, COX-2, and β-actin using TB Green^TM^ Premix Ex Taq^TM^ (Takara Bio Inc., Shiga, Japan). The primer sequences used in this study are summarized in Table 1. PCR reactions were performed in triplicate employing Thermal Cycler Dice^®^ Real Time System Lite (Takara Bio Inc., Shiga, Japan) using the following conditions: initial denaturation at 95 °C for 30 s, 40 cycles at 95 °C for 5 s, and 60 °C for 30 s; dissociation curve at 95 °C for 15 s, 60 °C for 30 s, and 95 °C for 15 s.

### 2.8. Histological Analysis

The colon tissues were fixed in 10% buffered-neutral formalin. The colon sections were subjected to alcian blue PAS staining to observe histopathological change (DKKorea, Seoul, Korea). Histological changes were examined by light microscopy. Microscopic fields for examination were chosen randomly and viewed at a magnification of 100×.

### 2.9. Gut Microbiota Analysis

Fresh fecal samples were collected from each mouse at the beginning and the end of the feeding trial in this study. Total DNA from the feces was extracted using QIAamp PowerFecal DNA kit (Qiagen, Venlo, The Netherlands) according to the manufacturer’s instruction. All DNA concentrations were measured using Qubit fluorometer (Invitrogen, Carlsbad, CA, USA) and adjusted to 5 ng/µL for MiSeq library construction using nuclease-fee sterile water. To investigate intestinal microbial community, MiSeq library construction was performed by two-step PCR according to the manufacturer’s instructions. Briefly, V4 hypervariable region of the 16S rRNA gene was amplified using, and the sequencing using 5 µL of forward primer (515F 5′-TCGTCGGCAGCGTCAGATGTGTATAAGAGACAGGTGCCAGCMGCCGCGGTAA-3′) and reverse primer (806R 5′-GTCTCGTGGGCTCGGAGATGTGTATAAGAGACAGGGACTACHVGGGTWTCTAAT-3′), 2.5 µL genomic DNA and 12.5 µL 2× KAPA HiFi HotStart Ready Mix (KAPABIOSYSTEMS, Cape Town, South Africa) with the following cycle: initial denaturation at 95 °C for 3 m, 25 cycles at 95 °C for 30 s, 55 °C for 30 s and extension at 72 °C for 30 s, and final extension 72 °C for 5 m. PCR products were purified using HiAccubead (AccuGene, Incheon, Korea) according to the manufacturer’s instructions. The 2nd PCR was performed in standard reaction contained 5 µL PCR amplicons, 5 µL index primers, 25 µL 2× KAPA HiFi HotStart Ready Mix (KAPABIOSYSTEMS) and 10 µL DNase free water with the PCR condition as follows: initial denaturation at 95 °C for 3 m, 8 cycles at 95 °C for 30 s, 55 °C for 30 s and extension at 72 °C for 30 s, and final extension 72 °C for 5 m, followed by PCR purification with HiAccubead (AccuGene). Final PCR products from each sample were pooled in a 1.5 mL e-tube, and the sequencing using MiSeq was performed at Macrogen Inc. (Seoul, Korea). The output data from Miseq were analyzed using MOTHUR software [22] and the removal process of erroneous sequences was performed according to MiSeq standard operating procedure (MiSeq SOP) guidelines. In short, paired-end sequences from MiSeq were assembled using ‘make.contigs’ and performed the alignment process using the SILVA database (version 132) [23], singletons were removed using ‘split.abund’, VSEARCH was used to detect chimeric sequences [24], taxonomic classification was performed using ‘classify.seqs’ based on the Ribosomal Database Project (RDP) database (version 16) [25], the chimeric and non-bacterial sequences were removed using ‘remove.seqs’, and operational taxonomic units (OTUs) were allocated using ‘opti.clust’ algorithm [26]. Non-metric multidimensional scaling (NMDS) analysis was done based on Bray-Curtis dissimilarity [27]. The Phylogenetic Investigation of Communities by Reconstruction of Unobserved States 2 (PICRUSt2) was used to predict the intestinal metabolic pathways [28].

### 2.10. Statistical Analysis

The results are expressed as the means ± standard deviation (SD). Analysis of molecular variance (AMOVA) was used to estimate significant difference of the gut microbiota in NMDS. Ellipses in NMDS were drawn with a 0.95 confidence level using R vegan package. Statistical significance was accepted for *p* values different predicted metabolic pathways and visualized for ALDEx2 effect differences >1 [29]. The physical parameters were compared using Student’s *t*-test. In addition, the differential abundance analysis was performed using linear discriminant analysis effect size (LEfSe) analysis based on Kruskal–Wallis (KW) sum-rank test [30]. ALDEx2 [31] was used to determine significantly increased and decreased metabolic activities (effect size > 1). Spearman rank correlation analysis was performed to investigate associations between LefSe-selected OTUs and ALDEx2-selected metabolic pathways. Statistical significance was accepted for *p* values < 0.05.

## 3. Results and Discussion

### 3.1. In Vitro Antioxidant Activity of PT Extracts

Free radicals generated by exogenous and endogenous factor play an important role in the development of diseases accompanied by inflammation of cells and animals [32]. However, antioxidants containing polyphenols and flavonoids contribute to the prevention and treatment of inflammation and disease caused by these free radicals. Furthermore, previous studies reported that PT extracts play a high radical scavenging activity due to high content of total polyphenols and total flavonoids [11,13]. These compounds strengthen the immune system and prevent some diseases such as hypertension, cancer, type 2 diabetes, and allergic disease [12,33,34,35,36]. Previous studies reported that PT extracts increased the immune system related to natural killer (NK) cell activity in clinical demonstration model [17]. Furthermore, PT extracts enhanced the secretion of cytokines in macrophages via activation of NF-kB pathway [16]. In this study, we evaluated the antioxidant capacity of PT extracts by total polyphenol content and free radical scavenging activity (Table 2). PT extracts showed high amount of total polyphenol content (32.3 ± 0.8 mg GAE/g). Furthermore, PT extracts exhibited 50% DPPH free radical scavenging potential at 798.4 ± 80 µg/mL. PT extracts showed low DPPH free radical scavenging activity although total polyphenol content was high amount. Corsetto et al. [37] have previously reported that high content of total polyphenols provides anti-inflammatory activities via scavenging reactive oxygen species (ROS) or cellular endogenous defenses via enhancing antioxidant, while showing the low antioxidant capability in DPPH radical scavenging activity. Previously, we have reported that the yield rate of PT extracts was 13% and the concentration of porphyra-334 in this PT extracts was 68.45 ± 20% mg/g [17]. The main component of PT extracts, porphyra-334, has been reported to control intracellular redox condition via anti-oxidant effect through enhancing glutamate-cysteine lipase and glutamate-cysteine ligase modifier subunit levels by activating Nrf2/Keap1 pathway [38]. Furthermore, Ryu et al. [39] also reported that porphyra-334 enhanced the antioxidant enzymes expression by regulating Nrf2 in early response. These results indicate that the high concentration of porphyra-334 combined with polyphenol contents in PT extracts supports their potential role of reducing inflammation induced by DSS as a natural antioxidant source. Moreover, This PT extract (500 mg/kg) was reported to have increased NK cell activities and immune stimulatory effects in humans and mice, respectively [17,20], suggesting that the main active compound, porphyra-334, is likely active in this concentration.

### 3.2. Effects of Differentiation of T and B Cells by PT Extracts

**Figure 1:**
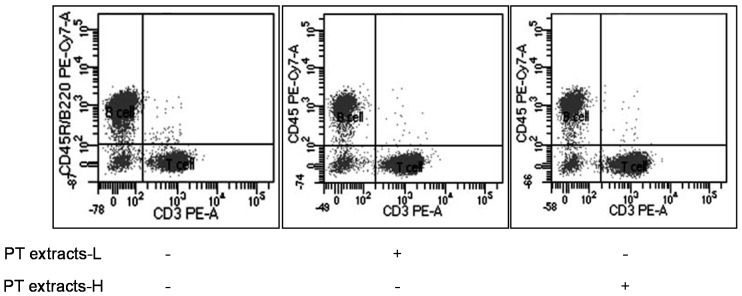
Effect of PT extracts on T cell population change. Flow cytometry analysis was performed to evaluate the effect of PT extracts on the populations of CD45R/B220^+^ B cells and CD3^+^ T cells.

Before the DSS treatment, we investigated the effects of low (500 mg of PT extracts/kg of body weight; PT extracts-L) and high (1000 mg of PT extracts/kg of body weight; PT extracts-H) concentration of PT extracts on mice immune system. PT extracts dissolved in normal saline and mice were intragastrically administered to mice. Flow cytometry was used to analyze the distribution of CD45R/B220^+^ B cells and CD3^+^ T cells in splenocytes (Figure 1). 

Table 3 showed that both high and low concentrations of PT extracts increased T cells approximately 20% without changing the ratio of Tc cell and Th/Treg cell. It has been reported that depletions of T cell and Treg cell were associated with severe mucosal injury [40,41,42,43], suggesting that the increased amount of T cells may play a role in alleviating damages caused by DSS-induced colitis. These results suggest that PT extracts changed the balance of the immune system by increasing the T cell population in mice, but it did not show a concentration-dependent pattern. Previously, Kang et al. [20] reported PT extracts showed no cytotoxicity in mice, while LDH assay showed dose-dependent cytotoxicity in PT extracts. As our results showed no significant difference between the concentrations of PT extracts (Table 3), we applied lower concentration of PT extracts for DSS-induced colitis experiments.

### 3.3. Inhibitory Effects of PT Extracts on DSS-Reduced Colitis Symptoms

**Figure 2:**
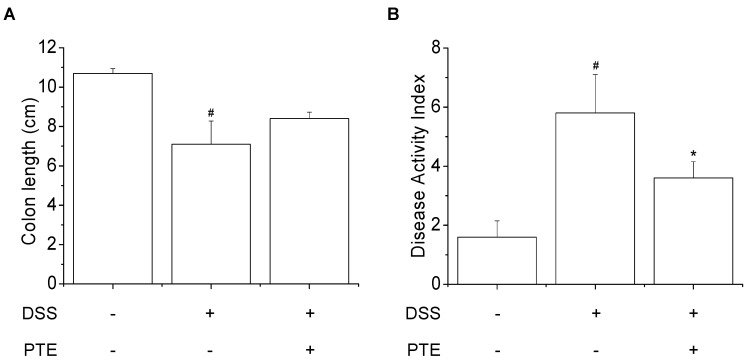
Effects of PT extracts on colitis development in DSS-treated mice. (**A**) Colon length in each group of the PT extracts treatments. Colon length was measured by ruler at the end of the experiment. (**B**) The disease activity index (DAI) score. The DAI is the sum of the three parameters such as body weight loss, stool consistency, and stool bleeding. All results are expressed as the means ± SD (*n* = 10). Compared with Control group, ^#^ *p* < 0.05, Compared with DSS-treated group, * *p* < 0.05. Control, DSS, and PTE indicated normal group, DSS-treated group, and DSS plus PT extracts-fed group, respectively.

Previous studies have shown that clinical symptoms of ulcerative colitis caused by DSS were body weight loss, colon length loss, and diarrhea [44]. In this study, we investigated the inhibitory effect of PT extracts on DSS-induced colitis symptoms in mice. While administration of DSS to mice significantly decreased their body weight, which was significantly ameliorated by feeding PT extracts (Table 4). DSS treatment also significantly shortened the length of the colon in DSS group compared to Control group (*p* < 0.05), which was slightly recovered by the PT extracts treatment (Figure 2A). In addition, increased DAI score by DSS treatment was significantly attenuated by PT extracts, suggesting that the clinical colitis symptom severity was effectively alleviated by PT extracts (Figure 2B). Moreover, DSS group exhibited severe colitis symptoms evidenced by the disappearance of goblet cells, superficial epithelial cells, and the increase of inflammatory cell infiltration in lamina propria and submucosa. PTE group showed that the histological damage was slightly alleviated (Figure S2B).

### 3.4. Inhibitory Effects of PT Extracts on DSS-Induced Inflammatory Markers

**Figure 3:**
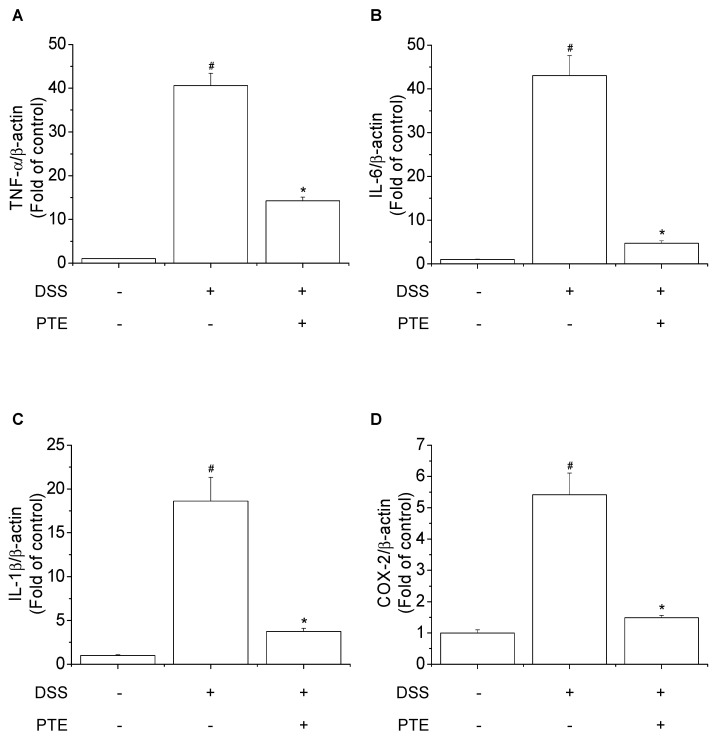
Effects of PT extracts on inflammatory markers in colon of DSS-treated mice. The mRNA expression of inflammatory markers was determined by real-time PCR primer of (**A**) TNF-α, (**B**) IL-6, (**C**) IL-1β, and (**D**) COX-2 in colon of DSS-treated mice. All results are expressed as the means ± SD (n = 10). Compared with Control group, ^#^ *p* < 0.05, Compared with DSS group, * *p* < 0.05. Control, DSS, and PTE indicated normal group, DSS-treated group, and DSS plus PT extracts-fed group, respectively.

Previously, the PT extracts used in this study were reported to enhance immune stimulatory effects through production of nitric oxide (NO) and expression of proinflammatory markers (IL-1β, IL-2, IL-4, IFN-γ, and iNOS) [20]. DSS is known to induce inflammation in the colon and play an important role in controlling the morphology of epithelial and goblet cells [45]. The inflammatory cytokine is known to reflect the degree of colonic inflammation by inducing inflammatory responses by DSS [46]. Previously, it has been reported that a toxicity of DSS increases the production of macrophage-derived cytokine such as IL-1β, IL-6 and TNF-α, and disrupts colonic epithelial cells [47]. To study the anti-inflammatory effect of PT extracts on DSS-treated mice, the mRNA expression of inflammatory markers in colon were determined. As shown in Figure 3, the mRNA expression of inflammatory markers (TNF-α, IL-6, IL-1β and COX-2) in mice colon was significantly increased after the treatment of DSS. However, the mRNA expression level of these inflammatory markers in the colon was significantly inhibited in colitis-induced mice administrated with PT extracts. It was reported that the extract from *P. yezonesis* reduced nitric oxide and reactive oxygen species as well as mRNA expression levels of inflammatory cytokines by controlling Toll-like receptor 4 (TLR4), which suppressed the activation of NF-κB and MAP kinases [48]. Recently, it has been reported that an increase of IL-6 leads to an inhibition response of Treg cell production, promoting the differentiation of T_0_ cell into Th17 cell [49,50]. Therefore, our results suggest that PT extracts treatment have shown anti-inflammatory activity on DSS-induced colitis mice via inhibiting inflammatory markers such as IL-6, leading to increase in T cell population.

### 3.5. Alteration of Gut Microbiota by PT Extracts on Colitis-Induced Mice

**Figure 4:**
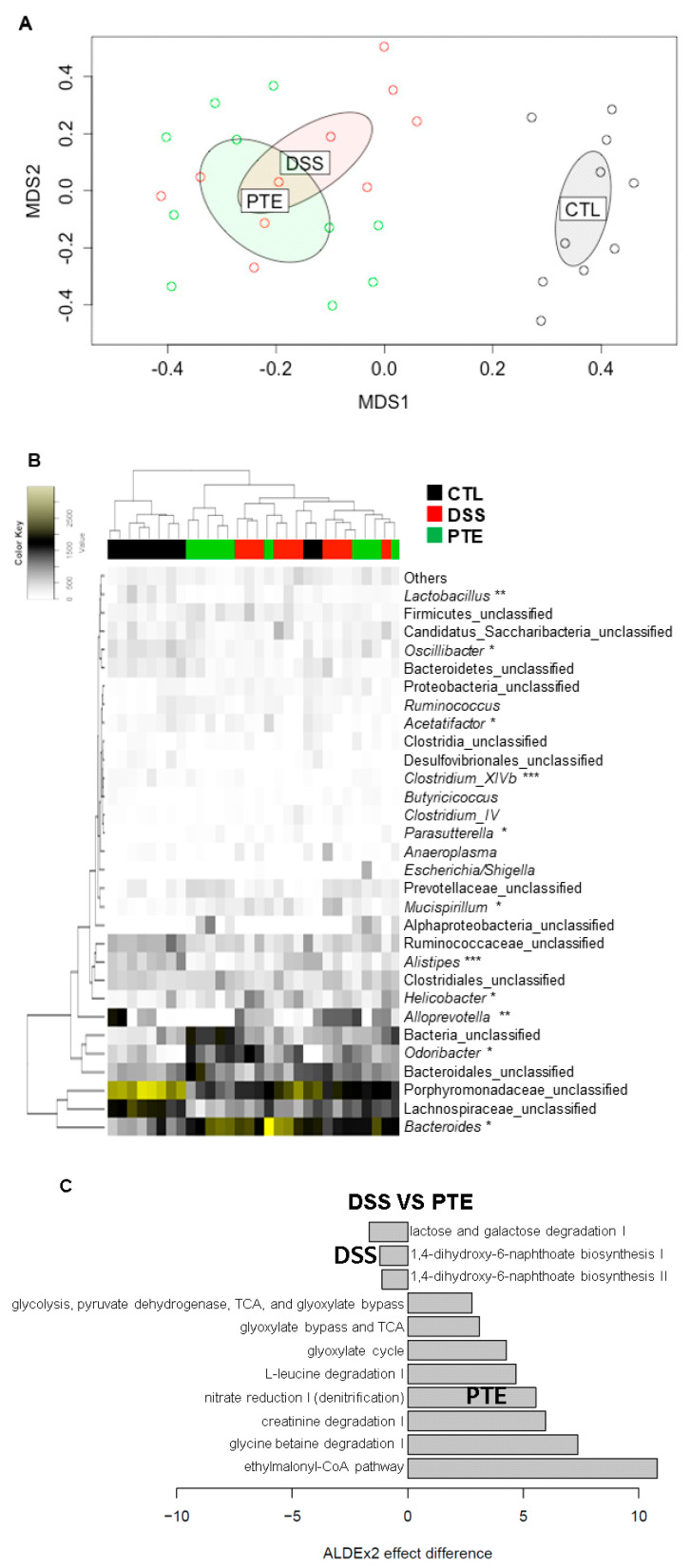
The comparison analysis of microbial community (*n* = 10) using non-metric multidimensional scaling (NMDS) plotting and heatmap. (**A**) NMDS analysis. The ellipses were drawn with 0.95 confidence level in NMDS plotting. (**B**) Genus level taxonomic composition of relative abundance in the mice gut microbiota. *, **, and *** indicate significant difference between CTL and DSS, DSS and PTE, and both, respectively. (**C**) Differentially abundant predicted metabolic pathways in DSS and PTE groups. CTL, DSS, and PTE indicate normal group, DSS-treated group and DSS plus PT extracts-treated group, respectively.

In non-multidimensional scaling (NMDS) analysis, the gut microbiota of CTL group was significantly different from DSS-treated groups (*p* < 0.001) (Figure 4A), indicating dysbiosis induced by DSS treatment. In addition, although the gut microbiota of DSS and PTE groups were close to each other in NMDS analysis, the results from AMOVA suggested that both DSS and PT extracts treatments significantly shifted the gut microbiota (*p* = 0.001). DSS treatment significantly decreased the abundance of the phylum Firmicutes (*p* < 0.001) and increased the genus *Bacteroides* (*p* < 0.01) (Figure 4B). There were 7, 2, and 2 genera whose abundance were significantly changed by DSS treatments, PT extracts, and both, respectively. Genera decreased by DSS include butyrate producing bacteria (i.e., *Alistipes*, *Oscillibacter*, *Acetatifactor*, and *Clostridium*_*XIVb*), while DSS increased the abundance of some of the potentially pathogenic bacteria (i.e., *Helicobacter*, *Mucispirillum*, and *Parasutterella*). On the other hand, PT extracts increased the abundance of *Clostridium*_*XIVb*, while decreasing the abundance of beneficial bacteria (i.e., *Alistipes* and *Lactobacillus*). Members of *Clostridium*_*XIVb* include cellulolytic bacteria and lactose-fermenting butyrate-producers [51]. Our results suggest that the increase in the abundance of *Clostridium*_*XIVb* may have affected the abundance of other polysaccharide utilizing bacteria such as *Alistipes* and *Lactobacillus*.

We also analyzed the effects of PT extracts on the intestinal metabolic pathways which were predicted based on gut microbial communities (Figure 4C). Among the predicted metabolic pathways, PT extracts depleted ‘lactose and galactose degradation’, allowing more bioavailability of lactose and galactose in gut. On the other hand, PT extracts enriched eight metabolic pathways in colitis-induced mice. Enriched glyoxylate cycle and ethylmalonyl-coA pathway may suggest that increased amount of succinate which can be used to produce short chain fatty acids, especially butyrate, in gut. Enriched nitrate reduction may suggest that increased nitrate bioavailability in gut. Hu et al. [52] reported that oral administration of nitrate regulated gut homeostasis and ameliorated DSS-induced colitis. Moreover, increased creatinine degradation indicates improved creatinine clearance and lower inflammation in renal fibrosis [53]. PT extracts enriched glycine betaine metabolism. It has been reported that administration of betaine inhibited inflammatory markers such TNF-α, IL-6, iNOS [54]. While results in Figure 4C suggest that PT extracts enhanced beneficial metabolic pathways in ameliorating DSS-induced colitis, PT extracts supplemented to non-DSS treated mice showed none of these beneficial effects (Figure S3). These results suggest that PT extracts may have ameliorated the damages caused by DSS-treatment through shifting DSS-induced gut dysbiosis. Together, our results suggest that PT extracts enriched beneficial metabolic pathways that may ameliorate adverse effects caused by DSS treatment. Intestinal bacteria produce butyrate through four pathways, namely acetyl-CoA, glutarate, 4-aminobutyrate/succinate and lysine pathways [55]. Among them, PT extracts enriched KEGG orthologues related to acetyl-CoA and 4-aminobytyrate pathways (Figure S4 and Table S1). It has been reported that Production of luminal butyrate by gut microbiota induce the differentiation of colonic regulatory T cells [56]. Therefore, our results indicate that PT extracts improve host’s immune system through shifting gut microbiota and their metabolic roles in gut.

In this study, we evaluated the effect of anti-inflammatory properties and gut microbiota modulation of *Porphyra tenera* (PT) extracts in DSS-induced colitis model. Previously, we reported PT extracts contain bioactive compound porphyra334 [20], approximately 16% of the total. Our results showed 500 mg/kg of PT extracts improved immune in DSS-treated mice, suggesting that 80 mg/kg of porphyra334 could bring the same beneficial effects. Further study should investigate the effects of porphyra334 for the development of functional food and supplements. The current study found that the PT extracts has beneficial effects on DSS-induced colitis disorders such as improving the body weight loss, colon length loss, and disease activity index (DAI) score of the colitis-induced mice. Moreover, PT extracts significantly restored the DSS-induced inflammation through enhancing the antioxidant activities in colitis mice. Importantly, we found that the PT extracts reshaped the gut microbes in DSS-treated mice. In this study, we did not evaluate oxidative stress nor expression of antioxidant enzymes. However, as increasing evidence suggested that DSS creates oxidative stress in mice [57] and induces pro-inflammatory cytokines [58]; thus, antioxidants are suggested to be used to treat IBD [59]. Moreover, a number of studies have reported that antioxidants alter cytokine production [60,61,62]. Our results showed antioxidant potential in PT extracts as well as reduction of pro-inflammatory cytokines by PT extracts. Therefore, it is likely that antioxidant activities of PT extracts ameliorated DSS-induced damages in mice.

## 4. Conclusions

Here, we conclude that PT extracts have potential antioxidants and anti-inflammatory properties as well as capabilities of modulating gut microbiota, which ameliorates DSS-induced colitis. Further studies, however, are required to confirm these in clinical trials.

## Figures and Tables

**Table 1:** Primer information for real-time PCR.

Gene	Forward Sequences	Reverse Sequences	NCBI Number
TNF-α	AGCCCCCAGTCTGTATCCTT	CATTCGAGGCTCCAGTGAAT	NM_013693.3
IL-6	AGTTGCCTTCTTGGGACTGA	CAGAATTGCCATTGCACAAC	NM_031168.2
IL-1β	GGGCCTCAAAGGAAAGAATC	TACCAGTTGGGGAACTCTGC	NM_008361.4
COX-2	AGAAGGAAATGGCTGCAGAA	GCTCGGCTTCCAGTATTGAG	NM_011198.4
β-actin	GGTGGGAATGGGTCAGAAGG	CAGCACAGGGTGCTCCTC	NM_007393.5

**Table 2:** Antioxidant capacities of PT extracts measured by total polyphenol content and DPPH radical scavenging assays.

Extracts	Total Polyphenol Content (mg GAE/g)	DPPH Radical Scavenging (IC_50_ µg/mL)
PT	32.3 ± 0.8	798.4 ± 80

Results represented as mean values from three repetition ± SD.

**Table 3:** The distribution of immune cells, including B cells and T cells, in splenocytes isolated from normal diet-fed mice.

Group	Live Cell	B Cell (CD45R/B220)	T Cell (CD3)	
			Tc Cell (CD8)	Th/Treg Cell (CD4)
Control *n* = 10	10,377 ± 55	5056 ± 179 (48.74%)	4595 ± 172 (44.27%)	
			1344 ± 52 (29.43%)	3151 ± 154 (68.41%)
PT extracts-L *n* = 10	9687 ± 16	2528 ± 91 (26.10%)	6424 ± 106 * (66.31%)	
			1804 ± 47 * (28.16%)	4499 ± 134 * (69.93%)
PT extracts-H *n* = 10	9635 ± 32	2788 ± 105 (28.93%)	6240 ± 156 * (64.75%)	
			1707 ± 44 * (27.44%)	4435 ± 158 * (70.97%)

Results are represented as the mean ± SD. * *p* < 0.05 represent significant increase compared to control. Control, Normal group; PT, *Porphyra tenera*; PT extracts-L, Low concentration of PT extracts (500 mg/kg)-treated group; PT extracts-H, High concentration of PT extracts (1000 mg/kg)-treated group.

**Table 4:** Effects of PT extracts on the body weight loss by DSS.

Group	D0	D7	Body Weight Loss
Control (*n* = 10)	21.6 ± 0.51	20.9 ± 0.32	0.8 ± 0.57
DSS (*n* = 10)	21.4 ± 0.73	19.0 ± 0.58	2.3 ± 0.15 ^#^
PTE (*n* = 10)	21.6 ± 0.61	20.3 ± 0.66	0.9 ± 0.34 *

Control, Normal group; DSS, Dextran sulfate sodium-treated group; PTE, PT extracts (500 mg/kg)-treated group. Compared with Control group, ^#^ *p* < 0.05, Compared with DSS group, * *p* < 0.05.

## Supplementary Materials

The following are available online at https://www.mdpi.com/2076-3921/9/10/988/s1, Figure S1: The sampling location of *Porphyra tenera* (Soan-do; 38°8′29.36″ N, 126°39′22.59″ E), Figure S2: Experiment design of animal experiment (A) and representative images of histological changes, Figure S3. Non-multidimensional scaling analysis showing gut microbiota difference at Day 0 (A) and Day 7 (B). Ellipses in NMDS were drawn with a 0.95 confidence level using R vegan package. Differentially abundant predicted metabolic pathways in CTL and NPTE groups (C). NPTE, PTE, DSS, and CTL indicate mice treated with PT extracts, mice treated with PT extracts and DSS, mice treated with DSS, and control mice not treated with PT extracts nor DSS, respectively. Figure S4: Significantly enriched and depleted KEGG orthologues (KO) in the butanoate metabolism, Table S1: The detail description of significantly enriched and depleted butanoate metabolic pathways.
