# Supplementary material for: Anti-Inflammatory Properties and Gut Microbiota Modulation of Porphyra tenera Extracts in Dextran Sodium Sulfate-Induced Colitis in Mice
Source: Antioxidants (Basel). 2020 Oct 14;9(10):988. doi: 10.3390/antiox9100988 (PMC7602078; doi:10.3390/antiox9100988)
Supplement: Supplementary file 1 [file antioxidants-09-00988-s001.pdf]

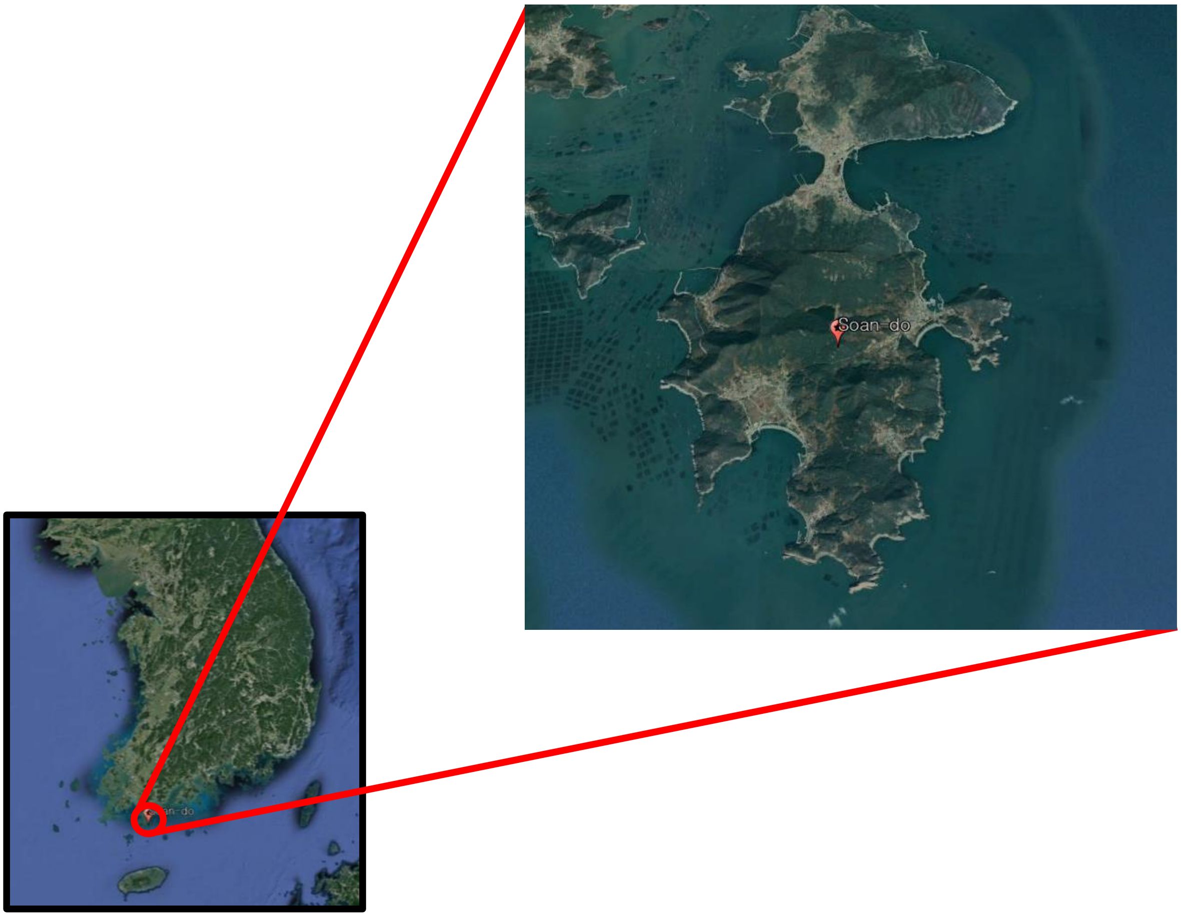

Figure 1. The sampling location of *Porphyra tenera* (Soan-do; 38°8′29.36"N, 126°39′22.59"E). These maps were modified using Google Earth software.

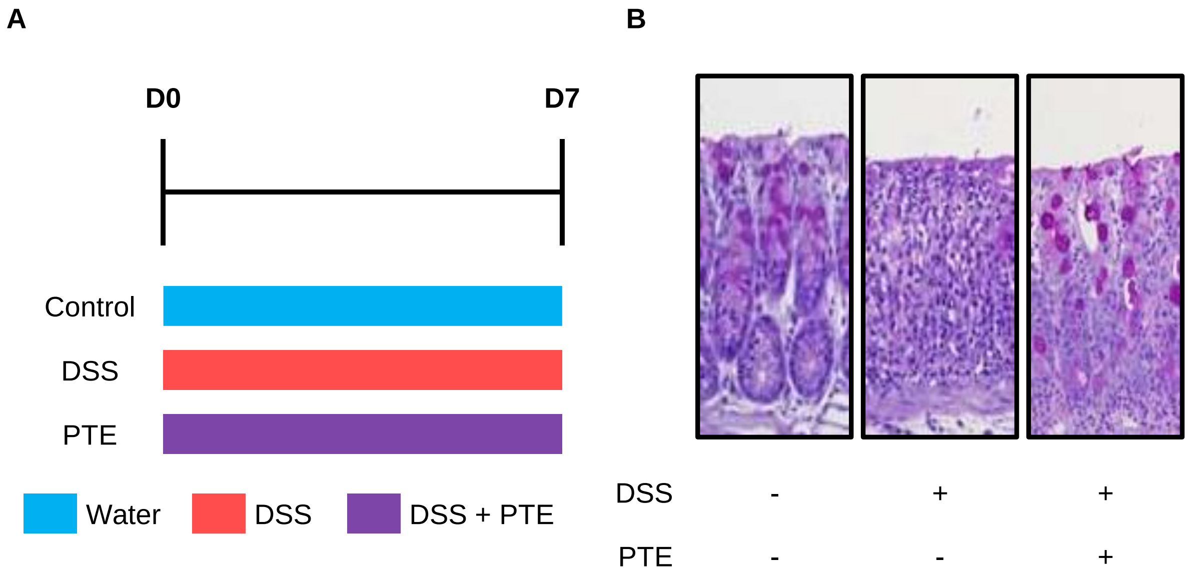

Figure 2. Experimental design of animal experiments (A) and representative images of histological changes (B). In histological analysis, alcian blue PAS staining was performed to evaluate the effect of PT extracts on the histological changes of goblet cells.

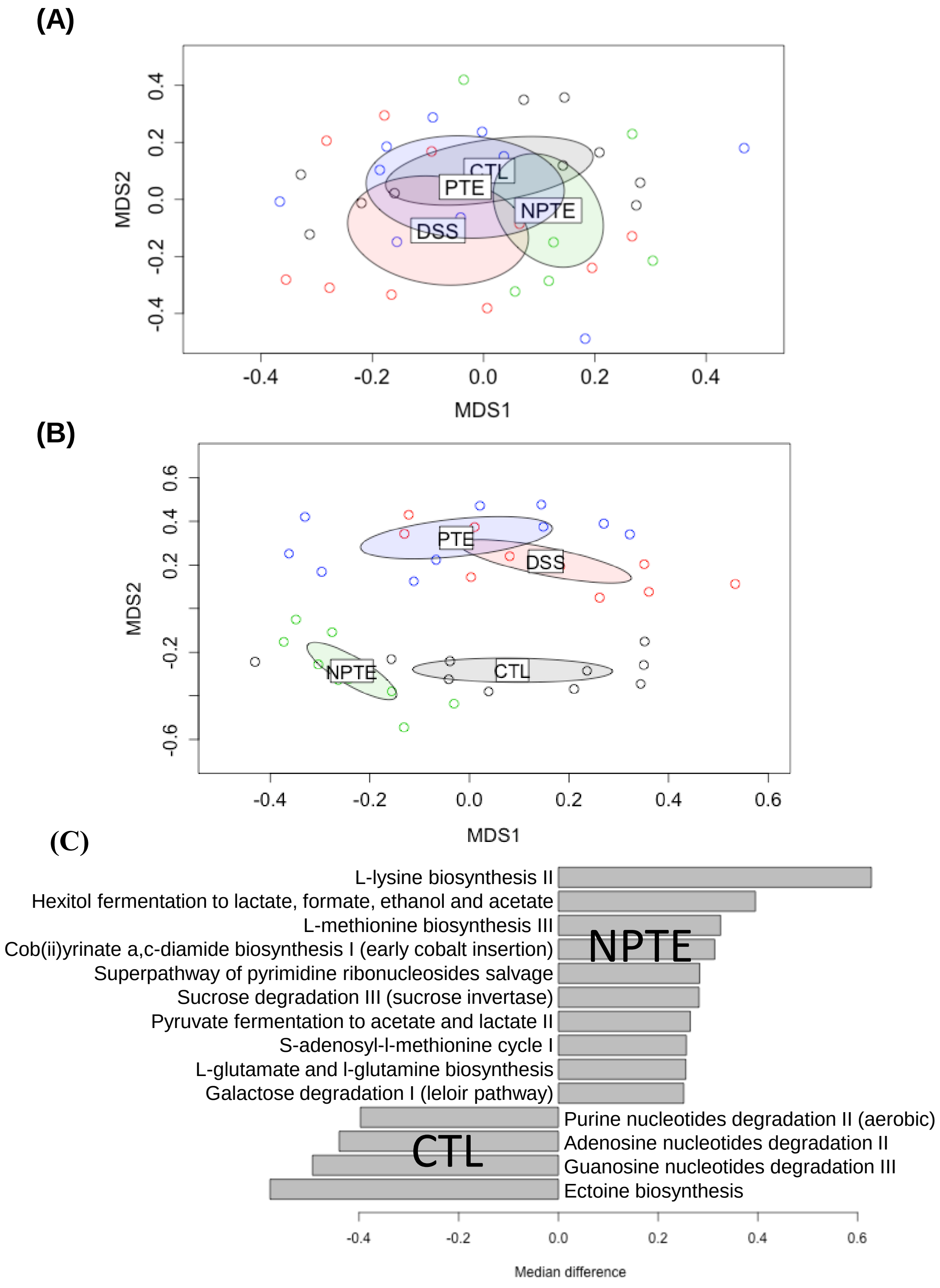

Figure 3. Non-multidimensional scaling analysis showing gut microbiota difference at Day 0 (A) and Day 7(B). Ellipses in NMDS were drawn with a 0.95 confidence level using R vegan package. Differentially abundant predicted metabolic pathways in CTL and NPTE groups (C). NPTE, PTE, DSS, and CTL indicate mice treated with PT extracts, mice treated with PT extracts and DSS, mice treated with DSS, and control mice not treated with PT extracts nor DSS, respectively.

Enriched

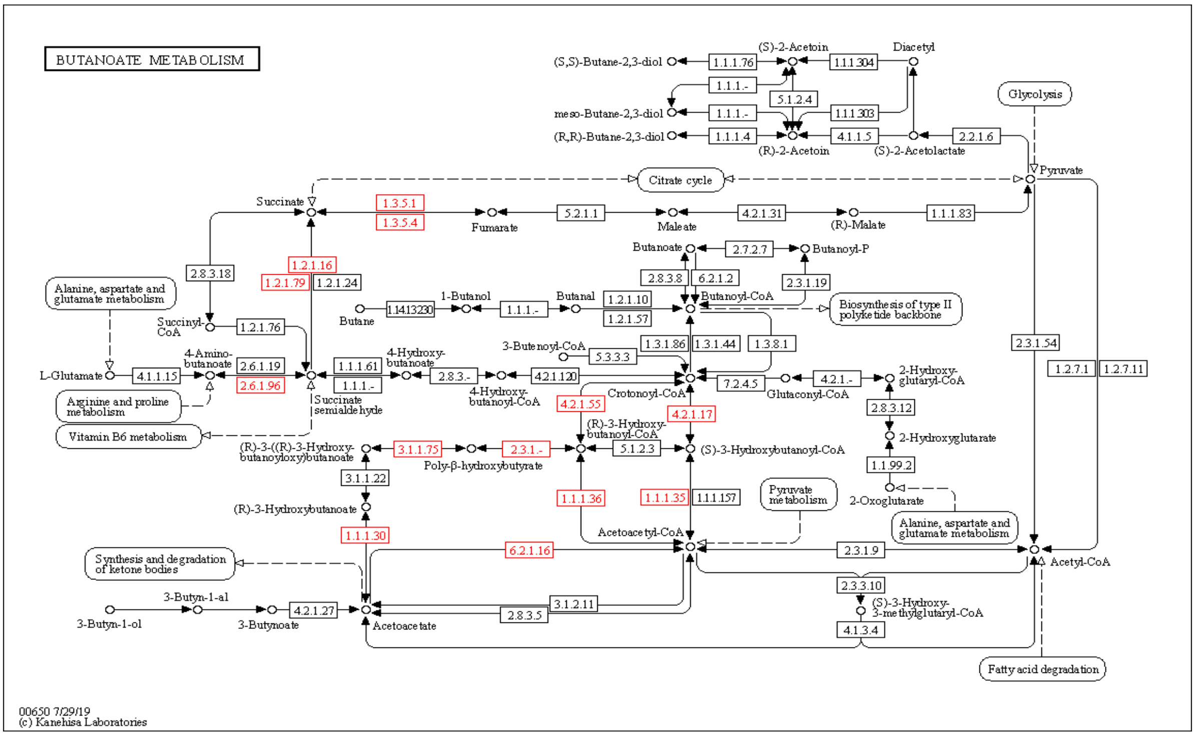

Depleted

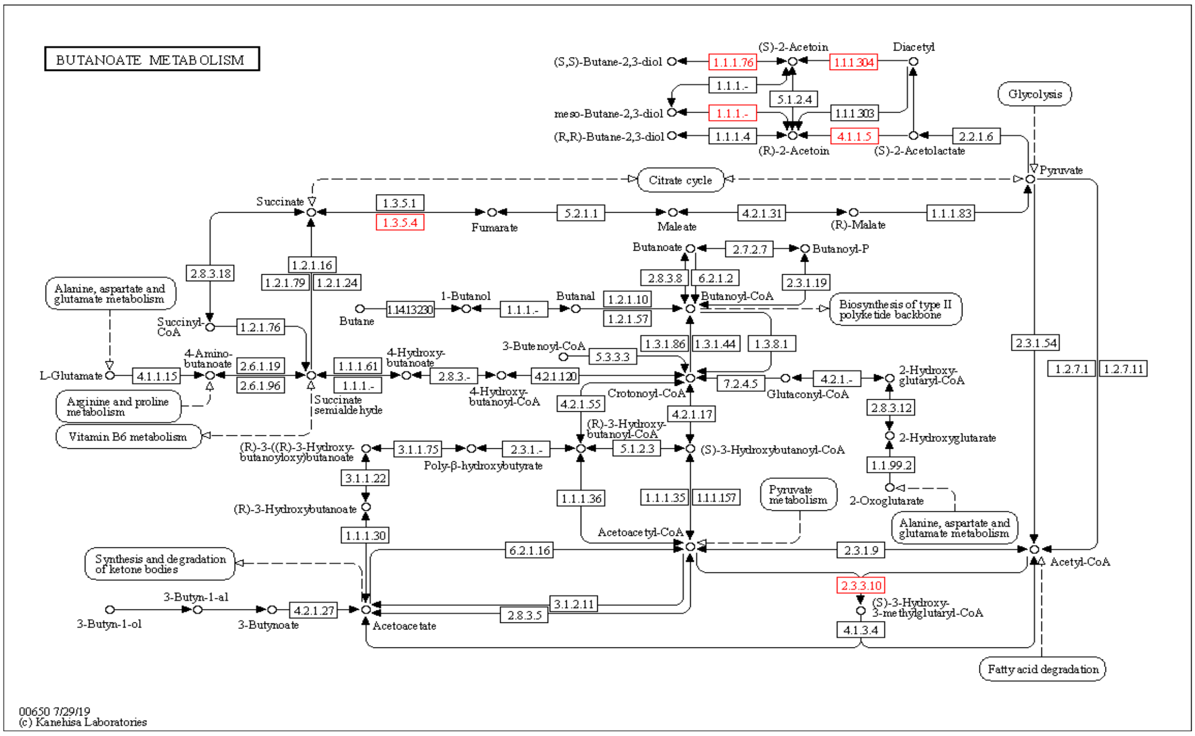

Figure 4. Significantly enriched and depleted KEGG orthologues (KO) in the butanoate metabolism.

Table 1. The detail description of significantly enriched and depleted butanoate metabolic pathways.

**KOs**[1]	**Name**	**Definitions**	**Effect size Difference**
K16871	POP2	4-aminobutyrate-pyruvate transaminase [EC:2.6.1.96]	2.309182
K01907	AACS	acetoacetyl-CoA synthetase [EC:6.2.1.16]	2.288249
K05973	phaZ	poly(3-hydroxybutyrate) depolymerase [EC:3.1.1.75]	2.037261
K03821	phbC	polyhydroxyalkanoate synthase subunit PhaC [EC:2.3.1.-]	1.652975
K00019	E1.1.1.30	3-hydroxybutyrate dehydrogenase [EC:1.1.1.30]	1.404745
K00242	sdhD	succinate dehydrogenase/fumarate reductase, membrane anchor subunit	0.994379
K17865	croR	3-hydroxybutyryl-CoA dehydratase [EC:4.2.1.55]	0.8773
K00135	gabD	succinate-semialdehyde dehydrogenase/glutarate-semialdehyde dehydrogenase [EC:1.2.1.16 1.2.1.79 1.2.1.20]	0.87691
K07516	fadN	3-hydroxyacyl-CoA dehydrogenase [EC:1.1.1.35]	0.774637
K00023	phbB	acetoacetyl-CoA reductase [EC:1.1.1.36]	0.653483
K01692	paaF	enoyl-CoA hydratase [EC:4.2.1.17]	0.592833
K00246	frdC	fumarate reductase subunit C	-0.56459
K00245	frdB	fumarate reductase iron-sulfur subunit [EC:1.3.5.4]	-0.62834
K03366	butA	meso-butanediol dehydrogenase/(S,S)-butanediol dehydrogenase/diacetyl reductase [EC:1.1.1.-1.1.1.76 1.1.1.304]	-0.6825
K01641	E2.3.3.10	hydroxymethylglutaryl-CoA synthase [EC:2.3.3.10]	-0.74624
K01575	alsD	acetolactate decarboxylase [EC:4.1.1.5]	-0.78032

[1]KEGG orthologues.
